# The Korea National Health and Nutrition Examination Survey (KNHANES): Current Status and Challenges

**DOI:** 10.4178/epih/e2014002

**Published:** 2014-04-30

**Authors:** Yuna Kim

**Affiliations:** Division of Health and Nutrition Survey, Korea Centers for Disease Control and Prevention

## OVERVIEW

The Korea National Health and Nutrition Examination Survey (KNHANES) is a national program designed to assess the health and nutritional status of adults and children in Korea. Since 1998, the KNHANES has collected data obtained by direct physical examination, clinical and laboratory tests, personal interviews, and related measurement procedures. These data are used to estimate the prevalence of chronic disease in the total population or monitor trends in the prevalence and risk behaviors.

## BRIEF HISTORY

The KNHANES was conducted on a triennial basis from 1998 to 2005. In 2007, the survey became a continuous, annual survey program conducted by the Korea Centers for Disease Control and Prevention (KCDC) and a variety of health measurements were added to the basic design to meet emerging data needs [1]. Since 2008, all health examination components have been conducted in the Mobile Examination Centers (MECs) that travel to each survey location. These MECs provide a standardized environment and equipment. In 2013, the computer-assisted personal interview system was introduced for both the health interview and nutrition surveys.

## SAMPLING METHODS

The KNHANES uses a complex, multi-stage probability sample design. The sample represents the total non-institutionalized civilian population of Korea. Since its development, the KNHANES sample design has changed to an on-going annual survey. Since 2007, every year and any combination of consecutive years comprise a nationally representative sample. A three-stage sample design is used for the KNHANES. The primary sample units (PSUs) are selected from a sampling frame of all census blocks or resident registration addresses. Each PSU consists of approximately 50-60 households. Following the selection of PSUs, all dwelling units in the PSU are listed and 20 households are selected through the field survey for household screening. The final stage of selection occurs in the household, where all members aged 1 year and over are selected to participate. Approximately 10,000 persons are sampled in total in all 192 PSUs per year. The expected total sample size is based on past KNHANES waves using the response rates for each subdomain of interest. The goal for the overall response rate for the KNHANES is 75%.

## SURVEY CONTENTS

Data on certain aspects of individuals’ lifestyles, such as smoking, alcohol consumption, physical activity, reproductive health, and dietary intake are collected. The diseases or medical conditions examined include obesity, hypertension, diabetes, hypercholesterolemia, chronic obstructive pulmonary disease, hepatitis, chronic kidney disease, oral health, anemia, arthritis, osteoporosis, thyroid function, and environmental exposures (e.g., lead, mercury, cadmium, cotinine). Depending on the age of the participant, the extent of examination differs. In general, blood and urine samples are collected from participants aged 10 years and over [2].

## SURVEY OPERATION

Health interviews and examinations are performed in the MECs and then the nutrition survey is conducted in the respondents’ homes a week later. The field operation team consists of nurses, a dentist, a radiological technician, interviewers, and dietitians. The MEC is open three days each week for 48 weeks of the year, with breaks of approximately two weeks each at New Years and during the summer. An advanced computer system with servers, notebook computers, and wide-area networking house all of the data. The operations staff can automatically transmit data into central databases. Survey information is available to KCDC staff within 24 hours of collection.

## QUALITY CONTROL

The objective of the KNHANES quality control program is to eliminate, control, and measure measurement errors. Non-sampling errors arise from numerous sources such as measurement and recording, recall problems, and interviewers’ mistakes. To reduce non-sampling errors, KCDC headquarters employs the following strategies: periodic staff training and field procedure monitoring by outside experts; feedback mechanisms; certification of examiners; use of a standardized environment; calibration of equipment on a regular basis; and comparison of findings over time. All laboratory samples are analyzed by a certified contract laboratory. The laboratory data quality control program monitors laboratory performance to ensure that all analytical values meet acceptable standards of precision and accuracy.

## DATA ANALYSIS

To produce unbiased cross-sectional estimates for the entire Korean population, the sample data can be inflated to the level of the population from which the sample is drawn. The most important consideration in analyzing KNHANES data involves taking into account the survey design. Survey sample weights should be used and the complex survey design must be accounted for in the estimation of variance [3]. The proper usage of sampling weights ensures that calculated estimates are truly representative of the Korean population. The sampling weights for each sample person are the product of three factors: the reciprocal of the probabilities of selection (PSU, household); an adjustment for non-response (household, person); and a post-stratification factor to make the resulting survey estimates for age, sex, metropolitan area, or province category approximately equal to the total population of Korea. To analyze samples over multiple years, sampling weights can be averaged over the sampled years. Age-adjustment is important to consider for trend analyses and comparisons between subgroups with different age distributions within KNHANES. The recommended standard population for comparison continues to be the projected population from 2005 by Statistics Korea.

## DATA DISSEMINATION

The KNHANES data are released for public use within one year of the end of each survey year. Documents such as the survey manuals and analytic guidelines are also provided to inform users of the limitations of the data. These are updated and expanded after each data release. Because the survey components and methods could vary with the year, all users, prior to any analysis of the KNHANES data, should read all relevant documentation on the survey. For users and researchers throughout the world, micro-data (in the form of SAS and SPSS files) and analytic guidelines can be downloaded on the KNHANES website in Korean (http://knhanes.cdc.go.kr/) free of charge. An English version of this website will be online by the end of 2014. Data that pose a risk of disclosure of the identity of a participant, such as the dates of data collection and detailed addresses, are not released to the public. These data are available only through the KCDC Division of Health and Nutrition Survey (DHANS). The DHANS provides on-site secure access to the full data set, while continuing to protect the confidentiality of respondents. In addition, the KCDC has organized an annual workshop designed to meet growing demands of KNHANES data users and promote broader, more proficient use of KNHANES data. Main workshop topics include how to prepare analytical data files such as locating variables of interest, merging data files, and using appropriate sample weights, as well as how to generate statistical estimates in SAS or SPSS. The 2014 KNHANES data analysis workshop is to be held on July 2. Participants need to register online through the KNHANES website (as shown above). Registration for the workshop will start in early June on a first-come, first-serve basis (visit the KNHANES website for more detailed information).

## VALUE OF THE KNHANES

The KNHANES is a survey of major importance to public health in Korea. It has achieved and maintained this position because of the breadth and depth of its data. The KNHANES has evolved to meet the health data needs of the nation. Its data are used to establish, develop, monitor, and evaluate national health programs and policies. The KNHANES produces objective, standardized data on the prevalence of a wide range of disease, conditions, and risk factors in the population. In addition, the KNHANES sets the standards for a large number of physiological measurements, including height and weight, body mass index, blood pressure, cholesterol, blood sugar, and bone density. KNHANES data indicate the average and distribution of these measures in the population, thus creating reference populations with which other researchers may compare their results. The current KNHANES is widely viewed as having the best collection of individual tests and measures in Korea, thereby making it perhaps the most effective survey overall.

## CHALLENGES AND THE FUTURE

The KNHANES faces challenges in its survey design and operations. First, the annual sample size is fixed due to financial and operational constraints, detailed demographic or geographic subgroups are too small to produce reliable estimates. Additionally, KNHANES respondents are not asked to participate in all survey components. For example, one-third of the total examined group is chosen at random to give heavy metals blood samples. Therefore, data users are cautioned to review sample sizes prior to attempting analyses previously performed by subgroups. With small sample sizes, analysts should consider combining subgroups. The KNHANES has been asked to oversample certain groups of particular interest for public health. The use of subgroup oversampling can ensure sufficient numbers, thus increasing the reliability and precision of estimates of health status indicators for these subgroups. Second, the KNHANES is designed as a cross-sectional study and longitudinal follow-up is limited to linkages of survey participants with National Health Insurance data and National Cancer Registry data. Interspersing cross-sectional and longitudinal components has been explored, but the effort to follow-up with the respondents was abandoned due to financial constraints and operational problems. Nonetheless, there is certainly strong scientific value in the longitudinal components and still a great deal of interest in designing future components of this nature. Finally, response rates have declined since 2010. There are challenges to obtaining or maintaining sample participation in each iteration of the survey, and a significant amount of staff time and resources are devoted to maintaining survey response rates. In a statistical sense, nonresponse can be considered either ignorable or nonignorable. If nonrespondents have significantly different characteristics from respondents, ignoring nonresponse leads to biased estimates. Methods of nonresponse bias analysis need to be developed and performed as soon as possible. However, the KNHANES budget is expected to be static over the next few years, and expenses will increase because of rising operating charges, meaning that there are no extra funds to meet these needs. This is a critical time for reviewing and rethinking the KNHANES. With the nation facing escalating chronic disease driven by the poor health habits of modern lifestyles, the KNHANES data will be even more useful in discovering intervention points and directing prevention strategies. The KNHANES has the scientific capacity to achieve this and more, but the survey needs to be supported at a level commensurate with its important mission.
